# Metal-Free Half-Metallicity in B-Doped gh-C_3_N_4_ Systems

**DOI:** 10.1186/s11671-018-2473-x

**Published:** 2018-02-20

**Authors:** Hailin Yu, Xuefan Jiang, Zhenguang Shao, Jinfu Feng, Xifeng Yang, Yushen Liu

**Affiliations:** Jiangsu Laboratory of Advanced Functional Materials, College of Physics and Electronic Engineering, Changshu Institute of Technology, Changshu, Jiangsu 215500 China

**Keywords:** Half-metallicity, Doped gh-C_3_N_4_, Strain effects, First-principles methods

## Abstract

Half-metallicity rising from the *s/p* electrons has been one of the hot topics in spintronics. Based on the first-principles of calculation, we explore the magnetic properties of the B-doped graphitic heptazine carbon nitride (gh-C_3_N_4_) system. Ferromagnetism is observed in the B-doped gh-C_3_N_4_ system. Interestingly, its ground state phase (B_C1_@gh-C_3_N_4_) presents a strong half-metal property. Furthermore, the half-metallicity in B_C1_@gh-C_3_N_4_ can sustain up to 5% compressive strain and 1.5% tensile strain. It will lose its half-metallicity, however, when the doping concentration is below 6.25%. Our results show that such a metal-free half-metallic system has promising spintronic applications.

## Background

Spintronic devices simultaneously utilize the charge and spin freedom of electrons and have attracted increasing attentions due to their potential use in logic and memory devices [1, 2]. Their performances, however, heavily depends on the spin polarization ratio of currents. There is a pressing need for materials that can generate 100% spin-polarized currents, therefore. Half-metal materials, which can do this at Fermi level *E*_*F*_, are considered as the ideal materials for spintronic devices [3–6]. Many half-metallic ferromagnets, such as doped manganites [7], double perovskites [8], and Heusler compounds [9, 10], have attracted extensive attentions in recent years. However, these half-metallic materials usually contain transition-metal (TM) and have strong spin-orbit coupling strengths, which result in short spin relaxation times. It is necessary to develop advanced TM-free half-metallic materials with long spin relaxation time, therefore.

Two-dimensional (2D) atomic crystals with planar surfaces have attracted a lot of attentions recently due to their potential application in spintronic devices [11–24]. Graphene and its several 2D analogues, such as hexagonal boron nitride and carbon nitride, have great potential for spintronics because of their exceptional properties, e.g., low dimensionality and electron confinement. Although most of these materials are non-magnetic in nature, there are many ways, such as doping and strain to reach the half-metallic ferromagnetism. For example, B, Al, and Cu embedded trizaine-based g-C_3_N_4_ (gt-C_3_N_4_) have been reported to be half-metallic [14]. The graphene-like carbon nitride also presents half-metallicity under tensile strain [17]. In addition, the heptazine-based g-C_3_N_4_ (gh-C_3_N_4_) has received a lot of attentions [25–33].

A large number of research works have investigated the electronic and magnetic properties of transition metal incorporated gh-C_3_N_4_ systems [11, 28, 30]. These transition metal embedded gh-C_3_N_4_ materials have been synthesized at elevated temperature [34–39]. Theoretical works show that the transition metals can bind more strongly with gh-C_3_N_4_ than with graphene and these systems are metallic [30]. Indrani et al. have systematically investigated the magnetic properties of C-dope gh-C_3_N_4_ systems by density functional theory (DFT) calculations [40]. They found that all of these C-dope gh-C_3_N_4_ systems are ferromagnetism, and a high energy phase shows strong half-metallicity and 400 K Curie temperature. Recently, Gao et al. [41] have experimentally demonstrated the capacity to fabricate the B-doped gh-C_3_N_4_ nanosheets, which present high-temperature ferromagnetism and half-metallicity. Despite of these early works, a systematic theoretical investigation of the B-doped gh-C_3_N_4_ is missing. Some fundamental issues such as the effects of doping position and B concentration on the electronic and magnetic properties of gh-C_3_N_4_ await clarification. Moreover, the effects of strain also need investigation.

In this work, we systematically investigate the effects of doping positions, B concentrations, and strain on the electronic and magnetic properties of the B-doped gh-C_3_N_4_ system through first-principles calculations. The results show that strong half-metallicity can be found in the ground state of B-doped gh-C_3_N_4_ (B_C1_@gh-C_3_N_4_). Not only doping positions but also doping concentrations play important roles in inducing half-metallicity. Moreover, the half-metallicity in B_C1_@gh-C_3_N_4_ can sustain up to 5% compressive strain and 1.5% tensile strain. The B-doped gh-C_3_N_4_ systems are promising for spintronics, therefore.

## Computation Methods

**Fig. 1:**
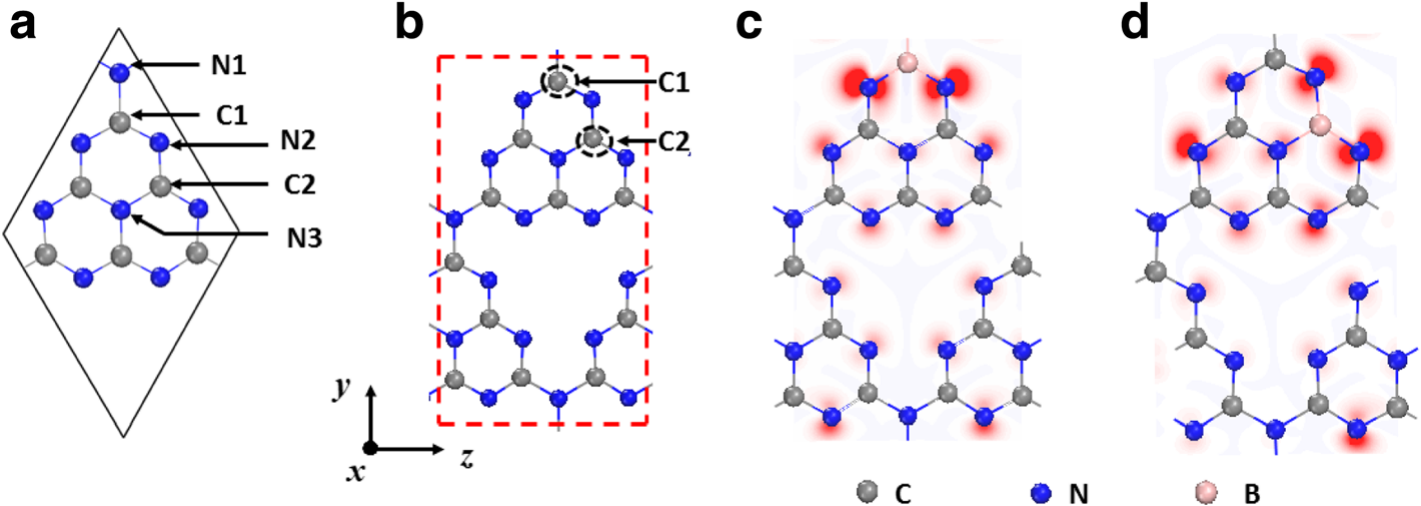
**a** Schematic representation of pristine gh-C_3_N_4_. There are two inequivalent C atoms (C1 and C2) and three inequivalent N atoms (N1, N2, and N3). **b** The tetragonal 28 a.u. cell of gh-C_3_N_4_ is used here to simulate the B-doped gh-C_3_N_4_ system (corresponding to 8.33% doping concentration). The black dashed circles indicate the possible B doping sites. **c**, **d** The optimized structures of B_C1_@gh-C_3_N_4_ and B_C2_@gh-C_3_N_4_, respectively. Distributions of charge density of spin-up state minus spin-down state for B_C1_@gh-C_3_N_4_ and B_C2_@gh-C_3_N_4_ are also shown here. The red and blue colors label the spin-up and spin-down charges, respectively

A tetragonal 28 a.u. cell containing two primitive cells of gh-C_3_N_4_ as shown in Fig. 1 has been employed to simulate the B-doped gh-C_3_N_4_ system. The geometry structure relaxation and static electronic structure calculation are performed by using the VASP package [42, 43], which is based on the density functional theory (DFT). The generalized-gradient approximation (GGA) of the Perdew–Burke–Ernzerhof (PBE) [44] and projector augmented wave (PAW) potentials are used. The cutoff energy is set at 500 eV and a 1 × 9 × 15 Monkhorst-Pack k-points grid is chosen to achieve a balance between the calculation time and the accuracy. All of the geometry structures are fully relaxed. The convergence threshold is set at 10^−6^ eV in electronic steps and 5 × 10^−3^ eV/Å in force. In order to avoid the interaction between two adjacent periodic images, the vacuum region along the *x*-direction is set at 15 Å. To investigate the effects of doping concentrations, a tetragonal 112-atomic supercell composed of 2 × 2 × 1 tetragonal unit cells and a 1 × 5 × 9 Monkhorst-Pack k-points grid are adopted.

## Results and Discussions

**Fig. 2:**
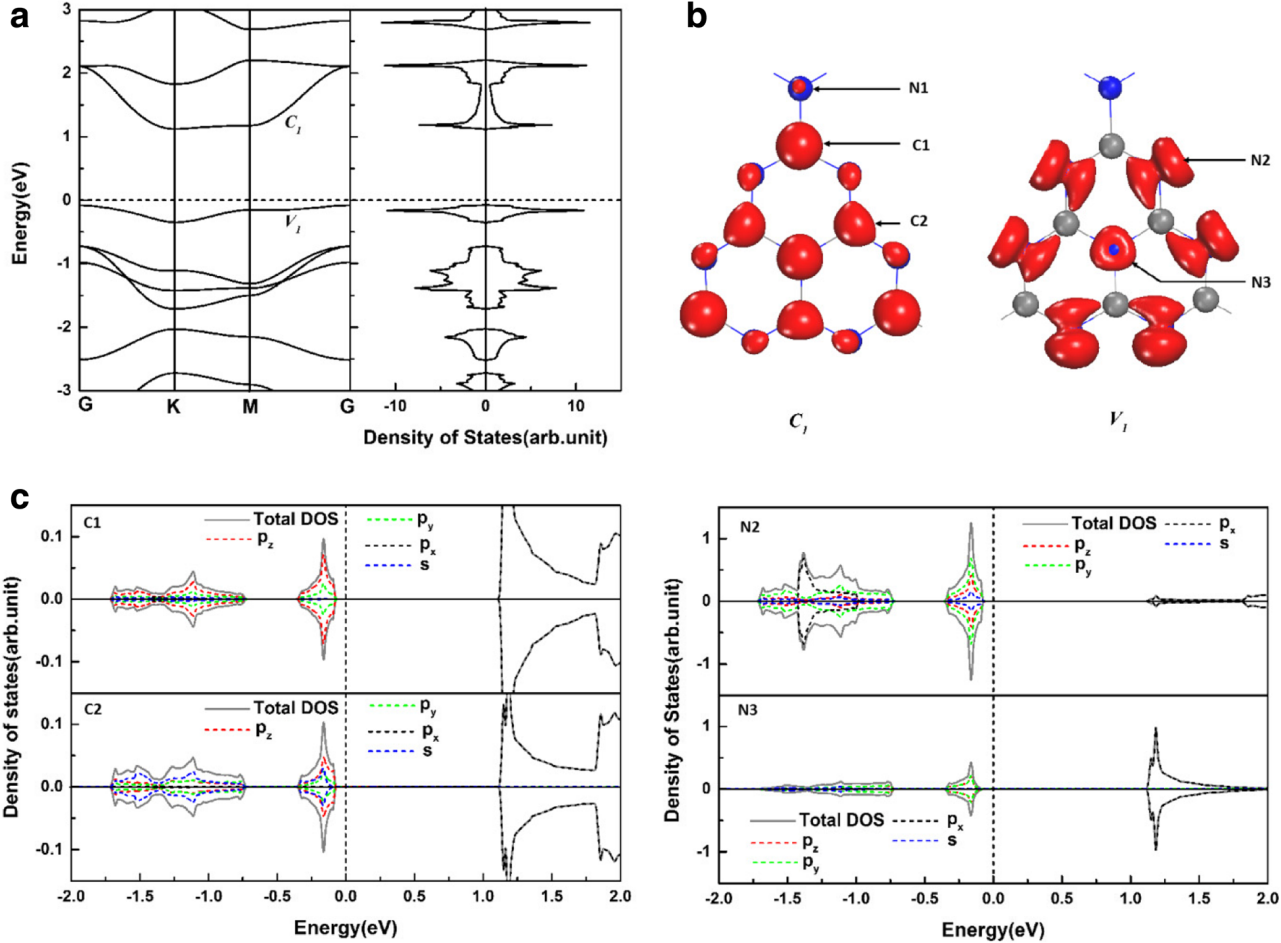
**a** The electronic band structures and the total density of states of pristine gh-C_3_N_4_. **b** The charge distributions of the edge bands *C*_*1*_ and *V*_*1*_ (indexed in **a**). **c** The orbital-resolved electron density of states projected onto C1 atom, C2 atom, N2 atom, and N3 atom (indexed in **b**). The energy at the Fermi level is set to zero

In a pure gh-C_3_N_4_ system, there are two inequivalent C atoms (C1 and C2) and three inequivalent N atoms (N1, N2, and N3) as shown in Fig. 1a. We find the relaxed lattice parameters (*a* = *b* = 7.14 Å) of the pure gh-C_3_N_4_ agree well with the previous experimental and theoretical reports [40, 45]. The band structure and the corresponding total density of states (DOSs) of gh-C_3_N_4_ are shown in Fig. 2a. To further understand the electronic properties of the gh-C_3_N_4_, the charge distributions of the edge bands *C*_*1*_, *V*_*1*_, and the corresponding local density of states are presented in Fig. 2b, c. It can be clearly seen that the bottom of conduction band *C*_*1*_ is dominated by the π^*^ states of C1, C2, and N3 atoms, which originate from the *p*_*x*_ orbitals. However, the top of valence band *V*_*1*_ is determined by the non-bonding δ states of N2 atoms and the π states of N3 atoms.

A tetragonal unit cell containing 28 atoms of gh-C_3_N_4_ (corresponding to 8.333% doping concentration) is employed to simulate the B-doped gh-C_3_N_4_ system as shown in Fig. 1b (the red dashed line). After considering early report [31] that the substitution on the C sites (C1 and C2) is more favorable than on the N sites (N1, N2, and N3), only the configurations of B substituting C have been investigated to explore their magnetic properties. As a result, the two different B-doped gh-C_3_N_4_ isomers (B_C1_@gh-C_3_N_4_ and B_C2_@gh-C_3_N_4_) are studied. The fully relaxed structures of B_C1_@gh-C_3_N_4_ and B_C2_@gh-C_3_N_4_ are given in Fig. 1c, d, respectively.

The structural stability depends on the extent of cohesive and the system with negative and large absolute cohesive energy has better stability. The cohesive energies (*E*_coh_) of B_C1_@gh-C_3_N_4_ and B_C2_@gh-C_3_N_4_ have been calculated by using

$$ {E}_{\mathrm{coh}}=\left[{E}_{\mathrm{tot}}-\sum {M}_i{E}_i\right]/M\left(i=\mathrm{C},\mathrm{N},\mathrm{B}\right) $$

where *E*_tot_ is the total energy of a B-doped gh-C_3_N_4_ system and *E*_*i*_ is the energy of an isolated atom for element *i* in the same cell. The *M*_*i*_ and *M* are the number of the *i*th species and the total number of atoms presented in the B-doped gh-C_3_N_4_ system, respectively. We find that the cohesive energies are − 6.107 and − 6.097 eV per atom for B_C1_@gh-C_3_N_4_ and B_C2_@gh-C_3_N_4_, respectively. Thus, the B_C1_@gh-C_3_N_4_ phase is energetically favorable. This conclusion agrees well with the previous work [31]. To further study the relative stability of the two B-doped gh-C_3_N_4_ systems, the cohesive energies of 2D C_2_N and gh-C_3_N_4_, which have been synthesized experimentally, are calculated and equal to − 6.813 and − 6.091 eV per atom, respectively. Interestingly, both B_C1_@gh-C_3_N_4_ and B_C2_@ gh-C_3_N_4_ have intermediate cohesive energies between C_2_N and gh-C_3_N_4_. Consequently, they should have intermediate structural and mechanical stability.

In order to determine the thermodynamic feasibility and the relative energy cost of B_C1_@gh-C_3_N_4_ and B_C2_@gh-C_3_N_4_ when compared to their pristine 2D analogues, the formation energies have also been calculated using

$$ {E}_f=\left\lfloor {E}_{\mathrm{tot}}-\sum {M}_i{\mu}_i\right\rfloor /M\left(i=\mathrm{C},\mathrm{N},\mathrm{B}\right) $$

where *E*_tot_, *M*_*i*_, and *M* are the same as those for the calculation of cohesive energy. *μ*_*i*_ is the chemical potential of the *i*th species. Here, graphene, rhombohedral boron and gaseous nitrogen are used to determine the chemical potentials *μ*_*C*_, *μ*_*B*_, and *μ*_*N*_, respectively. The calculated formation energies are 0.222 and 0.232 eV per atom for B_C1_@gh-C_3_N_4_ and B_C2_@gh-C_3_N_4_, respectively. As a comparison, the formation energy of gh-C_3_N_4_ is 0.293 eV per atom. In addition, the calculated *E*_*f*_ values of B_C1_@gh-C_3_N_4_ and B_C2_@gh-C_3_N_4_ are slightly lower than gh-C_3_N_4_, indicating these B-doped gh-C_3_N_4_ isomers can be fabricated. Indeed, the synthesis of B-doped gh-C_3_N_4_ has been reported [41].

In order to find out the magnetic ground states of B_C1_@gh-C_3_N_4_ and B_C2_@gh-C_3_N_4_, we have investigated the non-spin polarized (NSP), ferromagnetic (FM), and antiferromagnetic (AFM) states. The results show that FM state is the ground state for the two B-doped gh-C_3_N_4_ systems, and their magnetic moments are both 1.0 *μ*_*B*_ per unit cell as shown in Table 1. For further understanding of the magnetism of the two B-doped gh-C_3_N_4_ systems, the spin-dependent charge densities of B_C1_@gh-C_3_N_4_ and B_C2_@gh-C_3_N_4_ have been investigated and depicted in Fig. 1c, d, respectively. Slightly different from the C-doped gh-C_3_N_4_ systems in which the spin density is mainly located at the doped C-sites [40], the spin density of B-doped gh-C_3_N_4_ is mainly localized at the 2-fold coordinated N2 atoms, especially the N2 atoms adjacent to the dopant B atoms, as shown in Fig. 1c, d. Because B dopant has one less electron than the substituted C atom, a π defect is induced in B-doped gh-C_3_N_4_ system, resulting in 1.0 *μ*_*B*_ magnetic moment.

**Table 1:** The calculated cohesive energies, formation energies, and magnetic moments for B_C1_@gh-C_3_N_4_ and B_C2_@gh-C_3_N_4_

	*E*_coh_ (eV per atom)	*E*_*f*_ (eV per atom)	*M* (*μ*_*B*_per unit)
B_C1_@gh-C_3_N_4_	− 6.107	0.222	1.0
B_C2_@gh-C_3_N_4_	− 6.097	0.232	1.0

**Fig. 3:**
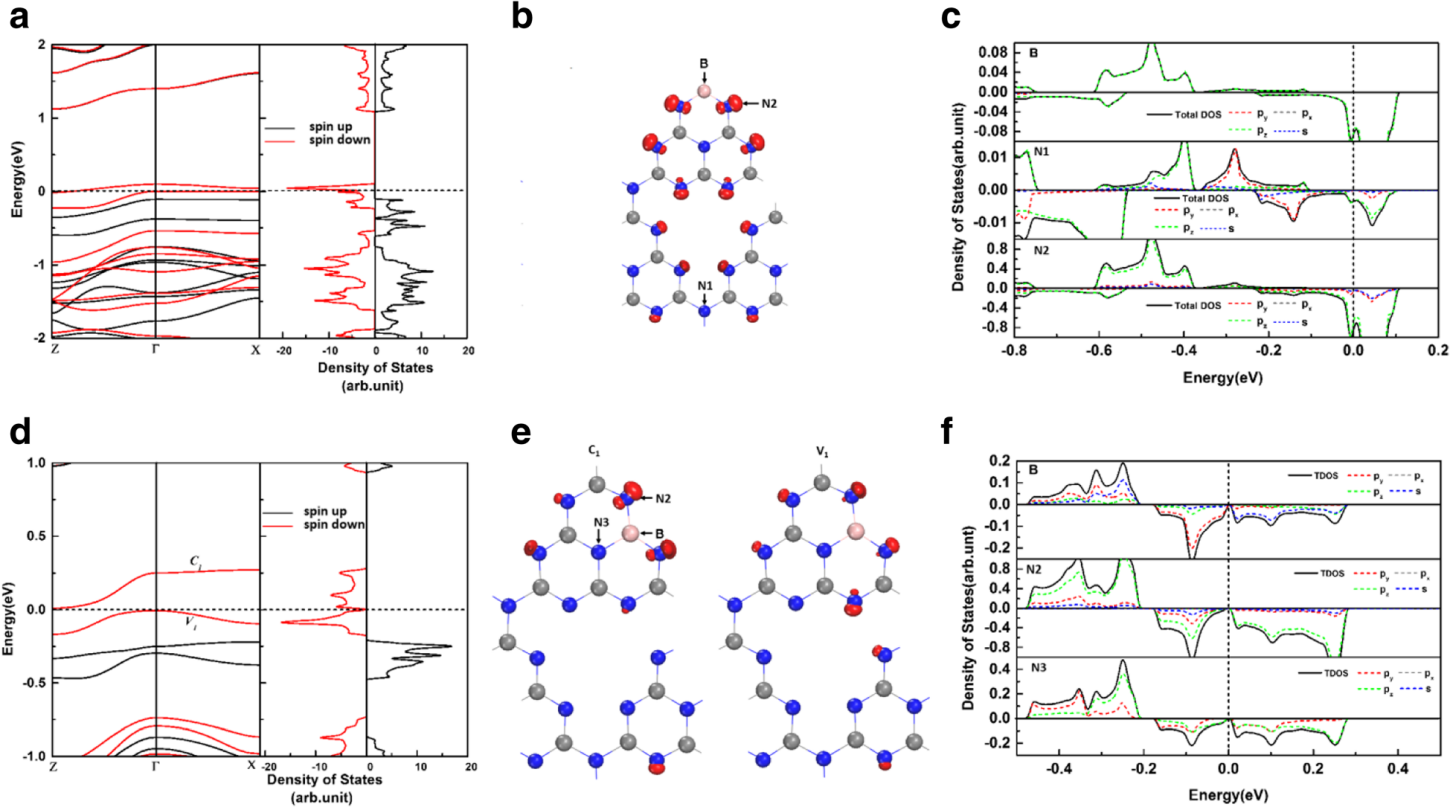
**a** The spin-dependent band structure and the total density of states of B_C1_@gh-C_3_N_4_. **b** The charge densities of the two bands crossing the Fermi level. **c** The orbital-resolved electron density of states projected onto B atom, N1 atom, and N2 atom (indexed in **b**) for B_C1_@gh-C_3_N_4_. **d**–**f** are the same with **a**–**c** but for B_C2_@gh-C_3_N_4_. The energy at the Fermi level is set to zero

To understand the effects of B doping on the gh-C_3_N_4_ systems, we performed the spin polarized band structure and density of states calculations for B_C1_@gh-C_3_N_4_ and B_C2_@gh-C_3_N_4_, as shown in Fig. 3a, d, respectively. The results show that the asymmetry between spin-up and spin-down densities in B_C1_@gh-C_3_N_4_ and B_C2_@gh-C_3_N_4_ induces an obvious magnetism. Interestingly, as shown in Fig. 3a, we find that the B_C1_@gh-C_3_N_4_ systems have a half-metallic property as one of the spin-channels is metallic, whereas the other one is insulating. The band structure and total density of states plots show that the spin splitting occurs close to the Fermi level and two spin-down bands are crossing the Fermi level, while the spin-up ones have a band gap of 1.23 eV. This is mainly because of the large voids present in the gh-C_3_N_4_ framework, which lead to the localization of electronic states. The band gap in the spin-up channel of B_C1_@gh-C_3_N_4_ is far larger than the gaps (in one of spin channel) of doped manganites [7], double perovskites [8], Heusler compounds [9, 10], and graphene nanoribbon [46]. The half-metallic strength of the B_C1_@gh-C_3_N_4_ systems can be comparable with the C-doped gh-C_3_N_4_ [40]. Such a strong half-metallic system is very promising because the spin-flip transition of carriers from the thermal excitation is not possible. To further explore the origins of the half-metallicity in B_C1_@gh-C_3_N_4_, the charge distributions of the two spin-down bands that across the Fermi level are presented in Fig. 3b. We clearly see that the half-metallicity of B_C1_@gh-C_3_N_4_ mainly comes from the non-bonding δ states of N2 atoms. The local density of states (see Fig. 3c) also shows that the half-metallicity of B_C1_@gh-C_3_N_4_ mainly originates from the *p*_*z*_ orbits of N2 atoms along with a partial contribution from the *p*_*z*_ orbits of B and N1 atoms. They are in good agreement with the earlier reports on gt-C_4_N_3_ [2], where the N orbitals provide a major contribution to the half-metallicity. For the B_C2_@gh-C_3_N_4_, the band structure and total density of states plots (Fig. 3a) also show that spin splitting occurs close to the Fermi level. The spin majority state has a band gap of 1.36 eV. However, the spin minority state shows a 0.016 eV band gap. The charge distributions of the edge bands and local density of states for B_C2_@gh-C_3_N_4_ show that both the valence band edges and the conduction band edges of B_C2_@gh-C_3_N_4_ are dominated by the non-bonding δ states, originating mainly from the *p*_*y*_ and *p*_*z*_ orbitals of N2 atoms. This means that the non-bonding δ states of N2 atoms are split when a B atom substitutes a C atom in gh-C_3_N_4_ system and determine its electronic properties.

**Fig. 4:**
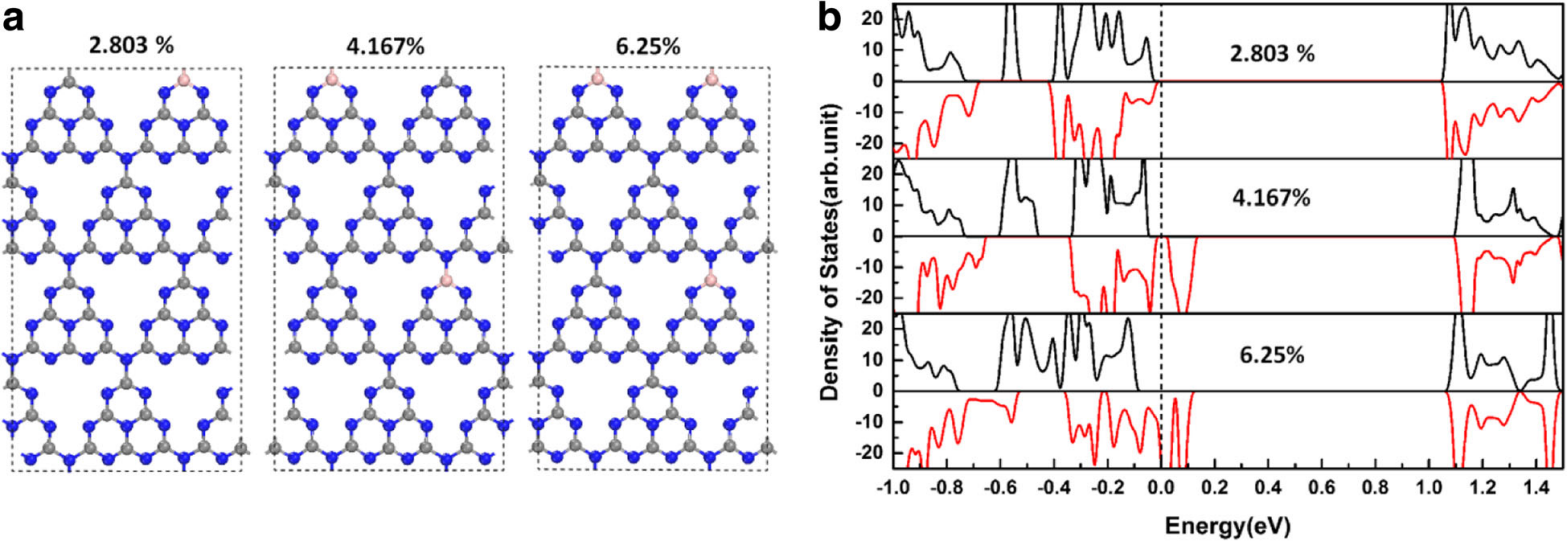
**a** Schematic representations of the tetragonal 112-atomic supercell used to simulate different doping concentrations of B_C1_@gh-C_3_N_4._ **b** The spin-dependent total density of states of B_C1_@gh-C_3_N_4_ with different doping concentrations. The energy at the Fermi level is set to zero

In order to clarify the dependence of half-metallicity in the B_C1_@gh-C_3_N_4_ systems on doping concentrations, a tetragonal 112-atomic supercell of 2 × 2 × 1 tetragonal unit cell has been employed and three different B-doping concentrations (2.083, 4.167, and 6.25%) are investigated, as shown in Fig. 4a, b. As we can see from Fig. 4b, B_C1_@gh-C_3_N_4_ can still sustain the half-metallicity for 6.25% doping concentration. However, it loses its half-metallicity as the doping concentration equal to or low than 4.167%.

**Fig. 5:**
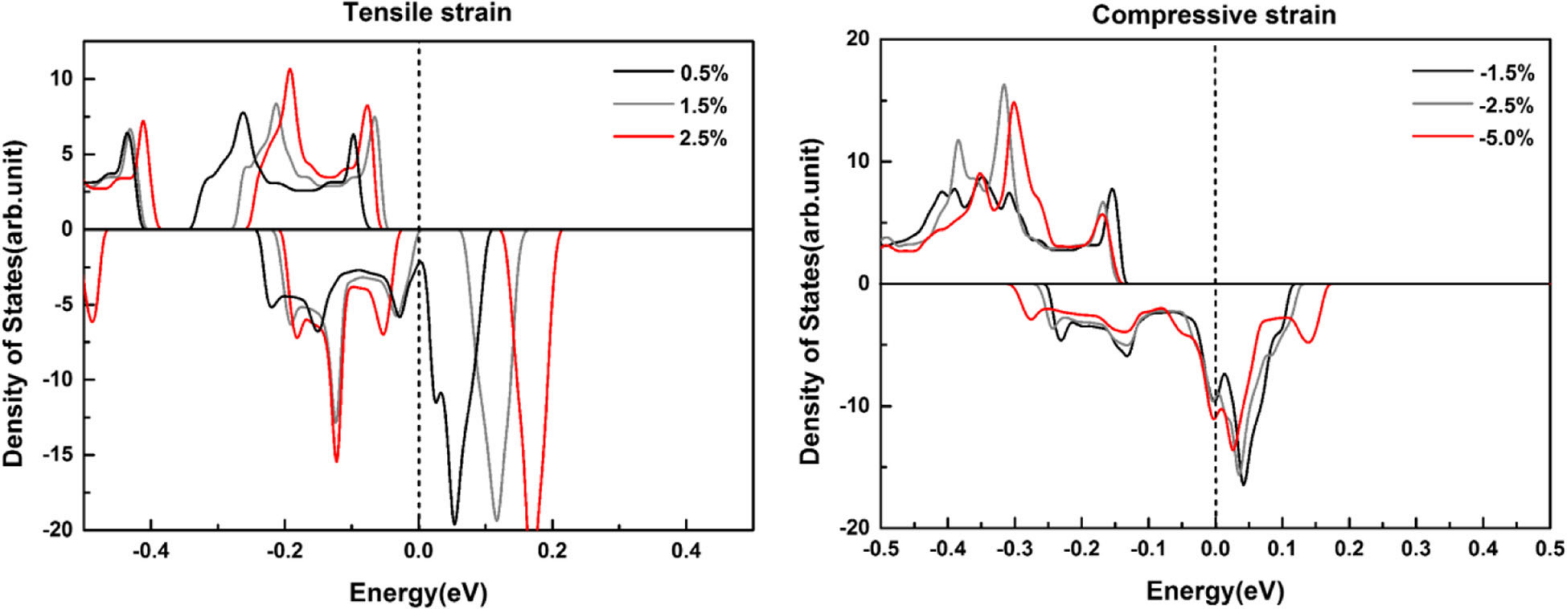
The spin-dependent total density of states of B_C1_@gh-C_3_N_4_ (with 8.33% doping concentration) under in-plain biaxial tensile strain (left) and biaxial compressive strain (right), respectively. The energy at the Fermi level is set to zero

Strain technology is commonly used to tune the spin properties of a magnetic material, and the strain effect on half-metallicity of a material should be studied. Here, we carried out the density of state calculations for the B_C1_@gh-C_3_N_4_ system under the in-plain biaxial strain. It is found that the half-metallicity strength gradually decreases as the biaxial tensile strain increases. It loses half-metallicity when the biaxial tensile strain reaches 1.5% as shown in the panel of Fig. 5. However, it sustains half-metallicity up to 5% of the biaxial compressive strain (see the right panel of Fig. 5). Thus, this system behaves well under external strain.

## Conclusion

Based on density functional theory calculations, the B-doped gh-C_3_N_4_ systems have been investigated for potential applications in spintronic devices. Ferromagnetism is observed in all B-doped gh-C_3_N_4_ systems. Moreover, a strong half-metallicity is achieved only in the ground state phase, i.e., B_C1_@gh-C_3_N_4_, which results from a spin split of the non-bonding δ states of highly unsaturated 2-fold coordinated N2 atoms. The half-metallicity is lost for low B-doping concentrations. Thus, both selective doping and its concentration play an important role in inducing magnetism and half-metallicity. The half-metallicity in B_C1_@gh-C_3_N_4_ can sustain up to 5% compressive strain and 1.5% tensile strain. These results show that the B-doped gh-C_3_N_4_ systems could be a ferromagnetic half-metallic material for magnetic memory and spintronic devices.
